# Assessment of Cognitive Function Among Pregnant Women With Anemia in the Early and Late Trimester of Pregnancy

**DOI:** 10.7759/cureus.74933

**Published:** 2024-12-01

**Authors:** Venugopal Lalitha, Rajendran Priyadharsini, Sathishbabu Murugaiyan, Parghavi Vaithiyanathan

**Affiliations:** 1 Physiology, Indira Gandhi Medical College and Research Institute, Puducherry, IND; 2 Pharmacology, Jawaharlal Institute of Postgraduate Medical Education and Research, Puducherry, IND; 3 Biochemistry, Jawaharlal Institute of Postgraduate Medical Education and Research, Puducherry, IND; 4 Medicine, Indira Gandhi Medical College and Research Institute, Puducherry, IND

**Keywords:** anemia, auditory, cognition, pregnancy, reaction time, trimester, visual

## Abstract

Background and aim: Cognitive development is an essential part of brain development. The cognitive assessment can be evaluated using the reaction time (RT) assessment. When attempting to comprehend cognitive processing and motor responses, RT is a very useful tool. Measuring RTs is an essential component in evaluating cognitive and motor abilities. The purpose of the current study was to assess the cognitive function of anemic pregnant South Indian women in the early and late trimesters of their pregnancy.

Methods: The present analytical cross-sectional study was conducted on 120 pregnant women in early and late pregnancy. A convenient sampling method was used. An RT machine assessed visual reaction time (VRT) and auditory reaction time (ART) in the subjects. All the data was tested for normality using the Kolmogorov-Smirnov test. Data analysis was done using a two-tailed unpaired Student's t-test.

Results: The distinction between pregnant women without anemia and those with anemia in terms of their VRT for red light (VRT-R) and green light (VRT-G) was highly significant with a p-value < 0.0001 (301± 8.16 Vs 324 ± 10.28 milliseconds). The differences in the ART for low-frequency sound (ART-low) and high-frequency sound (ART-high) were highly significant (214 ± 4.26 Vs 238 ± 5.38, 201 ± 2.08 Vs 222 ± 8.46 milliseconds). Pregnant women with anemia have significantly longer RTs compared to normal pregnant women in the early and late trimesters. There were significant differences in RTs between early and late trimesters in pregnant women with anemia, particularly for VRT-G (p< 0.0442) and ARTs (p< 0.0001 for ART-Low, p < 0.0416 for ART-High). The RTs improved slightly from the early to late trimester in pregnant women without anemia (VRT-R: p<0.002; VRT-G: p<0.0001; ART-low and high: p<0.0001).

Conclusion: Our study found a significant relationship between anemia in pregnancy and altered RTs. Anemia in pregnancy is associated with impaired cognitive function. Hence, pregnant women with anemia at different gestational ages are more prone to cognitive dysfunction than normal pregnant women. This highlights the importance of monitoring and managing anemia during pregnancy to mitigate its impact on cognitive functions.

## Introduction

Cognitive development is an essential part of brain development. The components of cognition include learning and memory, attention, concentration, language, and communication skills, which are interlinked [1]. The cognitive assessment can also be evaluated using one of the assessments, namely, the reaction time (RT). Any stimulus triggers a functional response in organs and tissues of the human body. The stimulus may be visual, auditory, gustatory, olfactory, or tactile. During the stimulation, deliberate, purposeful, and voluntary responses are generated. The delay between the introduction of a stimulus and the following response production to the trigger is known as RT.

Reaction time is an effective tool for understanding cognitive processes and motor responses. Its measurement is a critical component of cognitive and motor function evaluations [2]. An RT measures cognitive processing speed, which includes perception, memory, attention, decision-making, and motor response. It is commonly used in cognitive research to investigate attention, memory, executive functioning, and other cognitive tasks. Our human body responds to different stimuli at various levels and speeds. It responds faster to auditory stimuli than to visual stimuli. The RT is an index for information processing speed and the speed of response programming, which is a fundamental contributor to information processing [3].

Mehta et al. have reported an increase in auditory and visual RT in the first trimester of pregnancy [4]. Anemia during pregnancy carries the risk of premature birth, low birth weight, and fetus malformations [5]. Anemia is associated with impaired cognitive function [6]. Therefore, in the present study, we evaluate the cognitive function of South Indian pregnant women with anemia in the early and late trimesters of pregnancy.

## Materials and methods

This analytical cross-sectional study was conducted at the Department of Physiology, Indira Gandhi Medical College and Research Institute (IGMCRI), Puducherry, IND. After obtaining approval from the Institute Ethics Committee (approval no. IEC/PP/2016/31), a total of 120 pregnant women in the early and late trimester of pregnancy who visited the outpatient Department of Obstetrics and Gynecology at IGMCRI were recruited for the study.

A convenient sampling method was used. The pregnant women who visited the antenatal clinic were recruited from our tertiary care hospital. They were divided into two groups, namely the control group and the study group. The sample size was calculated based on a previous study [4]. A minimal sample size of 30 in each group was considered with an allowance for dropouts. The control group consists of 30 normal pregnant women (n = 60), and the study group consists of 60 newly diagnosed pregnant women with anemia (classification was based on the WHO criteria for anemia in pregnant women) in their early and late trimesters [7]. Pregnant women with hypertension, gestational diabetes mellitus, any other chronic medical ailment, multiple pregnancies, pregnant women currently taking iron treatment, upper limb abnormalities, and hearing and visual impairment were excluded from the research. After a light meal, participants were instructed to report to the physiology department's research laboratory at IGMCRI within two hours. The technique was properly explained to the participants, and they provided signed informed consent.

Reaction time measurement

Our human body responds to a wide range of stimuli with varying intensities and speeds. The subjects' visual reaction time (VRT) and auditory reaction time (ART) were measured using an RTM-608 machine (Medicaid Systems, Chandigarh, PB, IND) with a resolution of 0.001 seconds and an accuracy of ±1 digit. The machine used two distinct lights (red and green) and two different sounds (high and low pitch). The individuals were informed of the technique, and the tests were administered following sufficient training.

Visual Reaction Time

Each of the two lights, i.e., red and green, had a button equidistant from the central one. We examined the response to chromatic alterations while maintaining the same brightness for both hues. The participant was instructed to maintain the dominant hand's index finger on the center button and click the corresponding light button whenever a red or green light appeared. The response values were obtained directly from the digital display. Ten values of VRT were obtained; the two lowest and two highest values were eliminated, and the average of the middle six values was calculated. Visual reaction times in healthy adults are generally 0.25 seconds.

Auditory Reaction Time

Participants were instructed to maintain the dominant hand's index finger on the center button and hit the relevant volume button whenever a high-frequency or low-frequency beep sound was heard. The reaction values were directly read from the digital display. Ten values of ART were recorded; the two lowest and two highest values were deleted, and the average for the middle six values was calculated. The ART in healthy individuals is typically 0.17 seconds.

Statistical analysis

Data were entered in Microsoft Office Excel (Microsoft Corp., Redmond, WA, USA) and were presented as mean ± SD. The data followed a normal distribution and was analyzed using a two-tailed unpaired Student's t-test with GraphPad InStat version 3 (GraphPad Software Inc., San Diego, CA, USA) software. A p-value of less than 0.05 was considered statistically significant. 

## Results

Table 1 compares the age and hemoglobin levels of the control group and the study group in the early trimester. The difference in age between the two groups was statistically significant, with a p-value of 0.01. The control group has an average hemoglobin level of 12.98 g/dl ± 1.04. In contrast, the study group has a significantly lower average hemoglobin level of 8.92 g/dl ± 1.42.

**Table 1:** Comparison of age and hemoglobin status between the control group and study group in the early trimester of pregnancy Data are expressed as mean ± SD. Data analysis was done using the unpaired Student’s t-test. A p-value <0.05 was considered significant.

Parameters	Control group: Pregnant women without anemia (n = 30)	Study group: Pregnant women with anemia (n = 30)	p-value
Age (years)	21.14 ± 1.68	20.76 ± 1.66	0.01
Hemoglobin (g/dl)	12.98 ± 1.04	8.92 ±1.42	0.01

Table 2 compares the age and hemoglobin levels between the two groups of pregnant women (with and without anemia, respectively) in the late trimester. The difference in age between the two groups in the late trimester of pregnancy was statistically significant, with a p-value of 0.01.

**Table 2:** Comparison of age and hemoglobin status between the control group and study group in the late trimester of pregnancy Data are expressed as mean ± SD. Data analysis was done using the unpaired Student’s t-test. A p-value <0.05 was considered significant.

Parameters	Control group: Pregnant women without anemia (n = 30)	Study group: Pregnant women with anemia (n = 30)	p-value
Age (years)	21.84 ± 2.44	20.64 ± 2.72	0.01
Hemoglobin (g/dl)	12.06 ± 1.44	9.12 ± 1.56	0.01

Table 3 compares VRT and ART between the two groups of pregnant women in the early trimester. The difference in the VRT for red light (VRT-R) between the control group and the study group was highly significant with a p-value < 0.0001 (301± 8.16 Vs 324 ± 10.28 milliseconds). Similarly, the VRT for green light (VRT-G) between the two groups was also highly significant, with a p-value < 0.0001. The ART for low-frequency sound (ART-low) and high-frequency sound (ART-high) was highly significant (214 ± 4.26 Vs 238 ± 5.38, 201 ± 2.08 Vs 222 ± 8.46 milliseconds). This indicates that pregnant women with anemia have significantly longer RTs compared to normal pregnant women in the early trimester.

**Table 3:** Comparison of VRT and ART between the control group and study group in the early trimester of pregnancy Data are expressed as mean ± SD. Data analysis was done using the unpaired Student’s t-test. A p-value <0.05 was considered significant. VRT: Visual reaction time; ART: Auditory reaction time; VRT-R: Visual reaction time for red light; VRT-G: Visual reaction time for green light; ART-low: Auditory reaction time for low-frequency sound; ART-high: Auditory reaction time for high-frequency sound

Parameters (milliseconds)	Control group: Pregnant women without anemia (n = 30)	Study group: Pregnant women with anemia (n = 30)	p-value
VRT-R	301 ± 8.16	324 ± 10.28	<0.0001
VRT-G	310 ± 6.24	342 ± 8.72	<0.0001
ART-low	214 ± 4.26	238 ± 5.38	<0.0001
ART-high	201 ± 2.08	222 ± 8.46	<0.0001

Table 4 compares the VRT and ART between the control group and the study group in the late trimester. It was found that pregnant women with anemia have significantly longer reaction times compared to normal pregnant women in the late trimester concerning VRT-R, VRT-G, ART-low, and ART-high.

**Table 4:** Comparison of VRT and ART between the control group and study group in the late trimester of pregnancy Data are expressed as mean ± SD. Data analysis was done using the unpaired Student’s t-test. A p-value <0.05 was considered significant. VRT: Visual reaction time; ART: Auditory reaction time; VRT-R: Visual reaction time for red light; VRT-G: Visual reaction time for green light; ART-low: Auditory reaction time for low-frequency sound; ART-high: Auditory reaction time for high-frequency sound

Parameters (millisec)	Control group: Pregnant women without anemia (n = 30)	Study group: Pregnant women with anemia (n = 30)	p-value
VRT-R	296 ± 2.84	320 ± 5.10	<0.0001
VRT-G	302 ± 4.16	338 ± 6.12	<0.0001
ART-low	208 ± 2.14	232 ± 4.46	<0.0001
ART-high	198 ± 3.36	218 ± 6.24	<0.0001

Table 5 compares VRT and ART between early-trimester and late-trimester pregnant women with anemia. There were significant differences in RTs between early and late trimesters in pregnant women with anemia, particularly for VRT-G ( p< 0.0442) and ART (p< 0.0001 for ART-low, p < 0.0416 for ART-high).

**Table 5:** Comparison of VRT and ART between early-trimester pregnant women with anemia and late-trimester pregnant women with anemia Data are expressed as mean ± SD. Analysis of data was done using the unpaired Student’s t-test. A p-value <0.05 was considered significant. VRT: Visual reaction time; ART: Auditory reaction time; VRT-R: Visual reaction time for red light; VRT-G: Visual reaction time for green light; ART-low: Auditory reaction time for low-frequency sound; ART-high: Auditory reaction time for high-frequency sound

Parameters (milliseconds)	Early trimester with anemia (n = 30)	Late trimester with anemia (n = 30)	p-value
VRT-R	324 ± 10.28	320 ± 5.10	0.0612
VRT-G	342 ± 8.72	338 ± 6.12	0.0442
ART-low	238 ± 5.38	232 ± 4.46	<0.0001
ART-high	222 ± 8.46	218 ± 6.24	0.0416

Table 6 compares the VRT and ART between early-trimester and late-trimester pregnant women without anemia. It shows that reaction times improve slightly from early to late trimester in pregnant women without anemia (VRT-R: p<0.002; VRT-G: p<0.0001; ART-low and ART-high: p<0.0001).

**Table 6:** Comparison of VRT and ART between early-trimester pregnant women without anemia and late-trimester pregnant women without anemia Data are expressed as mean ± SD. Analysis of data was done using the unpaired Student’s t-test. A p-value <0.05 was considered significant. VRT: Visual reaction time; ART: Auditory reaction time; VRT-R: Visual reaction time for red light; VRT-G: Visual reaction time for green light; ART-low: Auditory reaction time for low-frequency sound; ART-high: Auditory reaction time for high-frequency sound

Parameters (milliseconds)	Early trimester without anemia (n = 30)	Late trimester without anemia (n = 30)	p-value
VRT-R	301 ± 8.16	296 ± 2.84	0.0024
VRT-G	310 ± 6.24	302 ± 4.16	<0.0001
ART-low	214 ± 4.26	208 ± 5.38	<0.0001
ART-high	201 ± 2.08	198 ± 3.36	<0.0001

## Discussion

In this study, we assess the cognitive function of pregnant women with anemia in different trimesters of pregnancy. Cognitive processes such as accuracy and RT are typically inferred from behavioral data. Reaction time is the key cognitive function essential in many occupations and daily living. It is one of the simplest and easiest ways to assess cognitive function. The findings of our study reveal that both visual choice [8] and auditory choice RT were increased in both early- and late-trimester pregnant women with anemia when compared with pregnant women without anemia. Similarly, Kolb et al. showed that iron status is a significant factor in cognitive performance in women of reproductive age. The severity of anemia primarily affects processing speed, and the severity of iron deficiency affects the accuracy of cognitive function over a broad range of tasks [9]. Thus, iron deficiency anemia in women is associated with impaired cognitive function, including slower RTs. This aligns with our findings that pregnant women with anemia have significantly longer VRTs and ARTs than those without anemia.

Another peculiar finding in our study was that RT was higher in the early trimester among pregnant women without anemia. This shows that the RT decreases as gestation increases, which means cognitive function is better in the late trimester. The same pattern was observed in the study group (pregnant women with anemia). This shows that cognition becomes better as pregnancy advances. This could be due to the effect of hormones affecting the pregnancy [10]. A study by Mehta et al. found that the rapid increase of human chorionic gonadotrophins during the first trimester could affect neuronal excitability, whereas the slow and persistent increase of estrogen and progesterone could have some effect on RT directly or indirectly [4]. Research done by Bruner et al. (1996) demonstrated that iron supplementation in anemic adolescent girls improved their RTs and cognitive performances [11]. Thus, anemia negatively affects RTs, as observed in our study. The probable reason is that the brain's energy metabolism and oxygen transport depend on iron. Low iron levels can cause brain tissues to get less oxygen, which can impede cognitive processes [12].

A study by Christensen et al. (2010) indicated that pregnancy itself can lead to changes in cognitive function, affecting RTs, and anemia exacerbates these changes. These findings are consistent with our findings that pregnant women with anemia have longer RTs than those without anemia [13]. Other studies have also shown similar results [14,15]. A study by Jelliffe-Pawlowski et al. (2006) found that cognitive function and RTs can vary across different trimesters of pregnancy [16]. Among the visual choice RTs, the RT was higher for green in both the control and study groups in the early and late trimesters of pregnancy. This shows that red is perceived better than green. Similarly, when comparing the auditory choice RT, the RT was less for high-frequency sound in both control and study group subjects in the early and late trimesters of pregnancy. Thus, treatment of anemia in pregnancy should be of major concern as the association between anemia, cognitive impairment, and mortality is higher in adults [17]. It has been found that mild anemia can substantially affect physical and cognitive capacities as well as the quality of life [18]. Hence, pregnant women, particularly those with anemia, should have their cognitive changes examined on a frequent basis to ensure prompt identification and management. Properly managing anemia through dietary changes, health supplements, and medical therapies can help reduce its influence on cognitive processes. Educating them about the cognitive changes that may occur during pregnancy and providing support might help them deal more effectively.

The strengths of our study include the comprehensive assessment of cognitive function across different trimesters, including the anemic and non-anemic pregnant women. The use of RT for cognitive evaluation is a straightforward measure to be done in a daycare setting. Our study limitations include assessing RTs in pregnant women with mild anemia and limiting the generalizability of the findings to those with moderate or severe anemia. Though the effect of hormonal influences was discussed, we did not measure the hormone levels, which would provide more insight into the impact on cognitive function. Future studies should include pregnant women with varying degrees of anemia to better understand the full impact on cognitive function. A larger sample size would enhance the generalizability of the findings. The use of a broader range of cognitive tests to capture different aspects of cognitive function beyond RT.

## Conclusions

Anemia in pregnancy is associated with impaired cognitive function. Hence, pregnant women with anemia are more prone to cognitive dysfunction than normal pregnant women. Early identification of anemia during pregnancy and prompt treatment with iron can reduce the incidence of cognitive impairment in these women and thereby reduce the risk of cardiovascular diseases. Prenatal care should take a holistic approach that considers both physical and cognitive health, ensuring that the mother and developing fetus get good care.
